# Severity-dependent atrial remodeling and atrial fibrillation vulnerability in a clinically relevant aortic regurgitation mouse model

**DOI:** 10.1172/jci.insight.200770

**Published:** 2026-03-12

**Authors:** Robert Lakin, Xueyan Liu, Dana Sherrard, Mihir Parikh, Ryan Debi, Nazari Polidovitch, Markus J. Duncan, Jian Wu, Peter H. Backx

**Affiliations:** 1 Department of Biology, York University, Toronto, Ontario, Canada.; 2 Department of Cardiology, China-Japan Union Hospital of Jilin University, Changchun, China.; 3 ParticipACTION, Toronto, Ontario, Canada.; 4 Shanghai Institute of Cardiovascular Diseases, Zhongshan Hospital and Institutes of Biomedical Sciences, Fudan University, Shanghai, China.

**Keywords:** Cardiology, Inflammation, Arrhythmias, Cardiovascular disease, Mouse models

## Abstract

Severity of aortic regurgitation correlates with atrial stretch and fibrillation. Uncovering stretch-mediated atrial remodeling prior to ventricular pathology will help define optimal timing of interventions

Atrial fibrillation (AF) affects 1%–2% of the population, with incidence rising steeply with age and adverse cardiovascular health (1). A population with one of the highest risks for AF is patients with mitral valve disease, especially when combined aortic valve stenosis, suggesting elevated ventricular filling pressures and atrial stretch may be central features in AF pathogenesis (2). A recent study reported a high AF prevalence in patients with aortic regurgitation (AR), with incidence tracking with regurgitation severity (3), fueling further speculation about the importance of stretch in AF (4). Remarkably, no preclinical studies have systematically quantified the dependence of AF (and atrial remodeling) on the degree of atrial stretch. Therefore, we created mice with graded levels of AR over a range that aligns with ACC/AHA guidelines to match the dependence of AF on AR severity in patients (5).

Regurgitation fraction was assessed by quantifying the ratio of the integrated diastolic backflow to systolic forward-flow (VTI_ratio_) across the aortic valve (Figure 1, A and B), with AR mice exhibiting a >90% survival rate (Supplemental Figure 1A). Immediately following valve disruption, we observed a restrictive flow pattern characterized by decreased E-waves coupled with increased A-waves (Figure 1C), which was accompanied by mild functional impairment (Supplemental Figure 1B; supplemental material available online with this article; https://doi.org/10.1172/jci.insight.200770DS1) and increased left ventricular (LV) end-diastolic pressures (LVEDPs) (Figure 1D). Whenever VTI_ratios_ exceeded approximately 50% early in the postoperative period, mice developed dyspnea. LVEDPs declined progressively during the first week following AR only to rise again thereafter (Figure 1E and Supplemental Figure 1C), with gradual reductions (*P* < 0.009) in ejection fraction (EF) and lusitropy (dPd*t*_min_) as well as increases (*P* ≤ 0.001) in LV end-systolic diameters (LVESD), end-diastolic diameters (LVEDD), and posterior wall thickness (Figure 1F and Supplemental Figure 1D). After 4 weeks, changes in LVEDD, LVESD, EF, and LVEDP tracked with AR severity (Figure 1G), mirroring the clinical progression in patients with AR (5).

After 4 weeks of AR, 10 of 16 mice developed AF, with AF durations correlating strongly with VTI_ratio_ (Figure 1, H–J). Atrial changes seen routinely in patients with AF such as fibrosis, hypertrophy, and macrophage (F4/80^+^) infiltration were observed in AR mice, which correlated (*P* ≤ 0.047) with VTI_ratio_ (Figure 1, K–M). Additionally, AR shortened (*P* < 0.001) both atrial effective refractory periods and action potential durations in isolated atria (Figure 1, N and O). Consistent with atrial hypertrophy and fibrosis, conduction velocity was slowed (*P* = 0.0004) in isolated atria from AR mice (Figure 1P), with evidence of sustained atrial arrhythmias (5/9, *P* = 0.002) consisting of (local and global) reentry, rotors, and conduction block, as observed in patients with AF (1) (Figure 1, Q and R).

By comparison, sustained arrhythmias were not seen in ventricles of AR (0/16) mice regardless of severity (Supplemental Figure 1E), consistent with the absence (*P* > 0.249) of fibrosis and macrophage infiltrations (Supplemental Figure 1, G–J, and Supplemental Table 1). Nevertheless, LV hypertrophy was observed in AR mice and correlated strongly with VTI_ratio_ (Supplemental Figure 1F), although hypertrophy was far less (*P* = 0.061) than in atria.

To gain deeper insight into molecular pathways driving AR-mediated cardiac remodeling, we performed qPCR mRNA measurements of genes associated with atrial fibrosis, inflammation, and hypertrophy observed in clinical AF (1). After 4 weeks of AR, we observed no increases in expression of collagen, inflammatory, or hypertrophic genes, despite overt atrial remodeling (Supplemental Table 2). However, because expression of fibrotic genes is increased relatively early compared to later in AF (6), when fibrosis may restrict atrial stretch, we measured mRNA levels after 1 week of AR. At 1 week, collagen I (*Col1a1*) and lysyl oxidase-like 2 (*Loxl2*) were increased in atria (Supplemental Table 2), consistent with enhanced collagen cross-linking in AF (7), whereas genes associated with tissue elasticity (*Col3a1*, *Fn1*, and *Loxl1*) were elevated in the LV (Supplemental Table 3). These findings underscore the complex transcriptional responses to AR and future studies will be needed to assess how hemodynamic loads and stretch affect the time-dependent fibrotic responses between the chambers.

Our mouse model recapitulates key features of human AR, thereby providing a platform for exploring the impact of AR severity on time- and chamber-dependent biochemical and cellular mechanisms underlying pathophysiological remodeling associated with myocardial stretch. Our studies establish that arrhythmogenic atrial remodeling appears well in advance of frank ventricular pathology, consistent with the high incidence of AF identified recently in patients with AR (3), which has been linked to atrial stretch induced by elevated filling pressures (4). Thus, the AR model offers new opportunities for identifying and exploring new strategies for the prevention and treatment of AF associated with volume overload.

## Data availability

Values for all data points in graphs are reported in the Supporting Data Values file.

## Conflict of interest

The authors have declared that no conflict of interest exists.

## Funding support

- Canadian Institutes of Health Research grant MOP-125950 (to PHB).
- Canada Research Chair in Cardiovascular Biology (to PHB).
- Canadian Foundation for Innovation John Evans Leader Award (to PHB).
- Canadian Institutes of Health Research Postdoctoral Fellowship (to RL).

## Supplementary Material

Supplemental data

Supporting data values

## Figures and Tables

**Figure 1:**
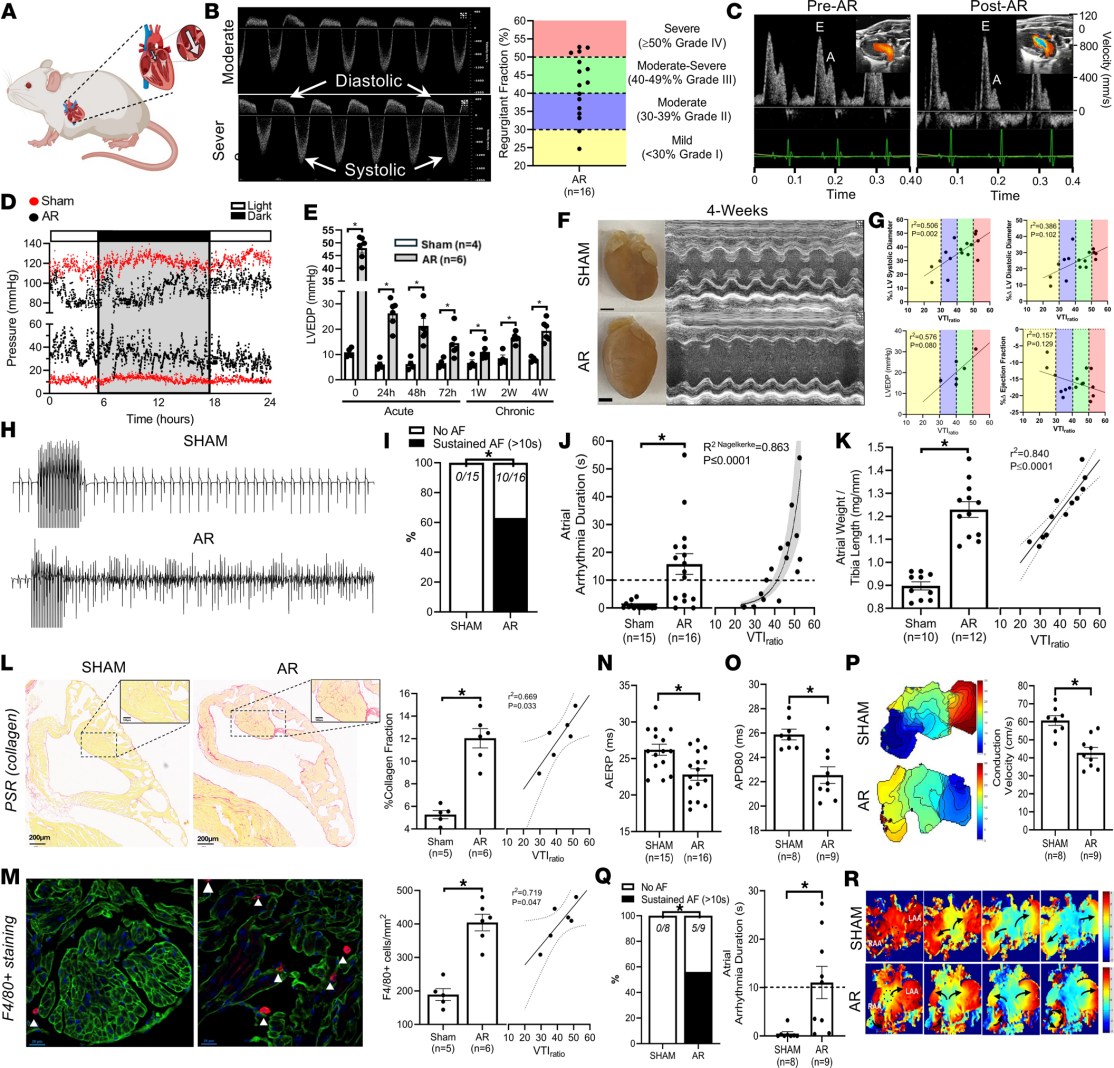
Graded aortic regurgitation (AR) induces atrial remodeling and atrial fibrillation (AF). (**A**) Schematic of AR generation. (**B**) Graded AR severities (VTI_ratio_) quantified in the aortic arch using Doppler ultrasound. (**C**) Post-AR mitral inflow changes consistent with restrictive left ventricular (LV) filling. (**D**) Twenty-four-hour LV systolic and diastolic pressures in Sham (red) and AR (black) mice; 12:12 light/dark cycles indicated. (**E**) LV end-diastolic pressure (LVEDP) increased immediately after AR, remaining elevated compared with Shams (2-way repeated measures ANOVA with Šidák’s multiple comparison test). (**F**) Gross morphology and LV M-mode 4 weeks after AR. Scale bars: 1 mm. (**G**) Changes in LV structural, functional, and hemodynamic indices with VTI_ratio_. (**H** and **I**) Atrial electrograms with programmed stimulations showing inducible AF in AR (10/16), but not Sham (0/15) mice. (**J**) AF durations increased with AR, correlating with VTI_ratio_ (gamma generalized linear model, 95% CI shown). (**K**) Atrial weight–to–tibia length ratio increased in AR versus Sham mice, increasing with VTI_ratio_. (**L**) Collagen deposition increased in left atrial appendages (LAAs) of AR versus Sham mice (left), with fibrosis increasing with VTI_ratio_ (right). Scale bars: 200 μm (×3 magnification) and 100 μm (insets) (×20 magnification). (**M**) Confocal LAA micrographs showing increased F4/80^+^ macrophage infiltration (arrows) in AR mice (left), with counts increasing with VTI_ratio_ (right). Scale bars: 20 μm (×40 magnification). (**N** and **O**) In vivo atrial effective refractory periods (AERPs) and ex vivo action potential durations (80% repolarization) (APD80) were shortened in AR mice. (**P**) Isochronal activation maps in paced (90 ms) atria revealed conduction slowing with AR. (**Q** and **R**) Ex vivo AF inducibility and optically mapped AF episodes were increased in isolated AR atria (5/9) but absent in Sham (0/8) mice. Data presented as mean ± SEM; *n* values indicated. *P < 0.05 by 2-tailed Student’s *t* test.

## Version 1 03/20/2026

In-Press Preview

## Version 2 05/08/2026

Electronic publication
