# Supplementary material for: Severity-dependent atrial remodeling and atrial fibrillation vulnerability in a clinically relevant aortic regurgitation mouse model
Source: JCI Insight. 2026 Mar 12;11(9):e200770. doi: 10.1172/jci.insight.200770 (PMC13235529; doi:10.1172/jci.insight.200770)
Supplement: Supplemental data [file jciinsight-11-200770-s002.pdf]

SUPPLEMENTAL MATERIAL

Severity-Dependent Atrial Remodeling and Atrial Fibrillation Vulnerability in a Clinically Relevant Aortic Regurgitation Mouse Model

Robert Lakin, Xueyan Liu, Dana Sherrard[#], Mihir Parikh[#], Ryan Debi, Nazari Polidovitch, Markus J. Duncan, Jian Wu, Peter H. Backx

#contributed equally and share 3rd authorship

SUPPLEMENTAL METHODS

Experimental animals: Male CD1 mice (7-8 weeks of age) from Charles River (Montreal, Canada) were used for all experiments. Mice were housed at a constant temperature (22±1°C) with a 12 h:12 h light-dark cycle and fed a standard laboratory mouse diet *ad libitum* with free access to water.

Aortic regurgitation surgery: Aortic regurgitation (AR) was induced under ultrasound echocardiography imaging guidance as previously described **(1)**. Briefly, mice were anesthetized with 3% isoflurane-oxygen mixture and administered Metacam (2mg/kg, s.c.) for analgesia. A midline incision was made, and the salivary glands were gently moved aside to locate and isolate right carotid artery from the vagus nerve. Next, a suture was tied anteriorly around the vessel with two additional threads, one acting as a bridge to prevent blood loss and the other to tie around the artery and secure a plastic micro-cannula containing a metal wire (Fine Science Tools, USA), were added posteriorly. Once inserted into the right carotid, the cannula was advanced from the proximal aorta to the aortic valve, and the metal wire was advanced through the cannula to

puncture the valve until significant peak retrograde diastolic flow (≥200 mm/s) was initially observed in the aortic arch using pulse-wave Doppler (Vevo2100, VisualSonics, Toronto, Canada)(see **Figure 1B**). Thereafter, systolic and diastolic aortic flows were quantified at the aortic arch using pulse-wave Doppler recordings. Systolic and diastolic velocity-time integrals (VTIs) were quantified in real-time by measuring the area of the negative ($VTI_{systolic}$) and positive ($VTI_{diastolic}$) flow profiles, with the ratio ($VTI_{diastolic/systolic}$) used to quantify the degree of regurgitation (VTI_{ratio}). To generate mice with increasing grades of AR, valve puncture was repeated and $VTI_{diastolic}$ was increased until the desired VTI_{ratio} was achieved. Sham-operated mice underwent the same procedure without puncturing of the aortic valve. To assess the nature of cardiac remodeling as a function of AR severity, only hearts with RegF ≥20% and ≤60% were included in the current study. Consistent with ACC/AHA Guidelines on AR **(2)**, the severity of AR was graded as follows: (Grade 1) Mild (<30% RegF); (Grade 2) Moderate (30-39% RegF); (Grade 3) Moderate-Severe (40-49% RegF); and (Grade 4) Severe (≥50% RegF).

A 4-week time period was chosen to align with the compensatory and asymptomatic phase of left ventricular remodeling (LV), prior to LV remodeling reaching functional thresholds (i.e., ejection fraction ≤55%) for surgical intervention as outlined in ACC/AHA guidelines **(2)**.

Telemetric Hemodynamics: Radiofrequency-emitting hemodynamic telemetry devices (PA-C10, Data Sciences International) were implanted in the left ventricle (LV) as previously described **(3)**. Briefly, mice were anesthetized (2.5% induction, 1.5%–2% maintenance isoflurane in oxygen), given a loading dose of Metacam (2mg/kg sc), and maintained at a temperature at 37˚C on a heating pad. Respiration was maintained at 1.5–2 Hz. Under aseptic conditions, the ventral

thoracic region was shaved, cleaned, and a ventral midline incision was made. The right common carotid artery was isolated with blunt dissection, ligated (5-0 silk), and bathed in 2% lidocaine solution. Following the Sham or aortic regurgitation procedure (described above), the hemodynamic catheter was quickly introduced into the carotid artery, advanced into the LV, and secured with suture. Correct positioning of the catheter within the LV was determined by monitoring the raw pressure trace to avoid contact pressures. Left ventricular end-systolic (LVESP) and end-diastolic (LVEDP) blood pressures were determined on a beat-to-beat basis and recorded continuously for the first week post-AR, and for a 48-hour window for each subsequent week thereafter. Data analysis was performed using Ponemah P3 Plus software (v6.4; Data Sciences International). Parameters were recorded at a sampling rate of 500Hz.

Echocardiography: Left ventricular (LV) functional and morphological remodeling in one-day Sham and AR mice was assessed as previously described **(4)**. Briefly, mice were anaesthetized with 1.5% isoflurane oxygen mixture and placed on a heated stage that maintained body temperature between 36.9-37.3°C. Transthoracic M-mode echocardiographic examination was conducted using an ultrasonic linear transducer scanning head (30MHz)(Vevo2100, VisualSonics, Toronto, Canada). Changes in LV structural and functional indices were assessed in the parasternal long-axis view using transthoracic M-mode. Ejection fraction (EF) was determined using bi-plane modified Simpson method (2D parasternal long-axis)**(5)**. Data analysis was performed using the VisualSonics cardiac data analysis suite (VEVO Lab).

Cardiac electrical remodeling and arrhythmia vulnerability: Electrical properties and arrhythmia vulnerability were assessed as previously described **(6)**. Briefly, following anaesthetization (1.5% isoflurane oxygen mixture), the right jugular vein was isolated, and a suture was tied anteriorly around the vessel. Next, a 2.0F octapolar recording/stimulation EP catheter (CI'BER Mouse, Numed) was inserted into the vessel and advanced into the right ventricle. Programmed electrical stimulations were delivered to the right ventricle to assess arrhythmia vulnerability. Our protocols for arrhythmia assessment were based on previously published protocols using burst pacing with intervals less than atrial effective refractory in mice **(7)**. All stimulations were delivered at a magnitude of 1.5x capture threshold and 1ms pulse duration. Effective refractory periods (ERPs) were determined by delivering nine pulses at a rate 20ms below the R-R interval followed by an extra stimulation. The S2 coupling interval was initially delivered above capture (~40ms) and reduced by variable increments until capture was achieved. For arrhythmia induction, two protocols were used. First, 27 pulses at 40ms intervals were applied the ventricle and reduced at 2ms decrements to 20ms. Next, 20 trains (every 1.5s) of 20 pulses (2ms duration) at a 20ms interpulse interval were applied. Only reproducible episodes of rapid, chaotic, and continuous ventricular activity ≥10s were defined as a sustained arrhythmic event.

Optical mapping: Optical mapping of isolated denervated atria was assessed as previously described **(8)**. Briefly, heparinized mice were euthanized with an anaesthetic overdose of isoflurane (~5%). After deep anaesthesia was achieved, the thorax was opened by mid-sternal incision. The heart was quickly excised into warn (35°C) Tyrodes solution (in mmol/L): 140 NaCl, 5.4 KCl, 1.2 KH_2PO_4, 1 $MgCl_2$, 1.8 $CaCl_2$, 5.55 D-glucose, 5 HEPES, and 10 U/mL heparin (pH 7.4). The heart was pinned to a Sylgard coated Petri dish, and the pericardium and any other residual tissue were excised. The atria were separated from the ventricles by making an incision

along the connective tissue in the atrioventricular groove. Atria were pinned to reveal the mitral and tricuspid valves, and atrial fat pads and residual tissue were removed. Next, the orientation of the atria was flipped to expose the pulmonary veins, the mitral and bicuspid valves were removed, and any additional residual tissue was excised. Finally, incisions were made in a straight path along the superior and inferior vena cava to 'open' the atria. Atria were then transferred to a separate dish and superfused continuously with carbogenized (95% O_2/5% CO_2) Krebs solution containing 20 mM 2,3-butanedione monoxime (BDM)(Sigma-Aldrich, B0753), to inhibit contraction and minimize movement artefacts **(9)**, from a reservoir container connected via a water-jacketed perfusion line to a perfusion pump (Masterflex® C/L® Analog Variable-Speed Pump) at a constant volume and flow rate (2.0-2.5 ml/min) at 35°C (in mmol/L): 118 NaCl, 4.2 KCl, 1.2 KH_2PO_4, 1.5 $CaCl_2$, 1.2 $MgSO_4$, 2.3 $NaHCO_3$, 20 D-glucose, and 2 Na-pyruvate (pH 7.35–7.4).

After atria were mounted and perfused, 3 Ag/AgCl electrodes were positioned within 1 mm in a lead II electrogram configuration. Electrodes were attached to a Biopac amplifier (UIM-100C, CA., USA) whose output was digitized (AXON CNS Minidigi 1B, Molecular Devices, CA., USA) and displayed continuously to record spontaneous rhythmic activity. If sinus rate dropped below 250 bpm, experiments were immediately terminated, and the heart was excluded from subsequent analyses.

Following 10-15 minutes of stabilization, isolated atria were stained for 5-7 min with a "loading" solution consisting of Kreb's with 20 mM BDM plus voltage-sensitive dye (10μM, Di-4-ANEPPS, Sigma-Aldrich) and then continuously superfused with carbogenized Krebs solution (35°C, pH: 7.35–7.4). Optical recordings were made by exciting the atrial surface using a high-powered LED illumination system (LEX20LZ4, 530 nm peak wavelength) controlled by an electronic shutter. Light was passed through a band-pass filter (531±40 nm) and fluorescent light

was passed through a 610 nm long-pass filter (Semrock, Rochester, NY). Images were collected from a 14 x 14 mm field of view using a 0.63x objective lens (NA = 0.35) and projected onto a complementary metal oxide semiconductor (CMOS) camera equipped with sensors containing 100x100 pixels (MiCAM Ultima-L, SciMedia, Costa Mesa, CA., USA).

Optical recordings were made during sinus rhythm or pacing at a 90-ms cycle length by applying 1ms pulses at a voltage 1.5x the capture threshold applied to the epicardial surface using platinum electrodes (spaced ~1 mm apart) attached to a stimulator (Pulsar i6 Stimulator, Frederick Haer & Co (FHC), Bowdoinham, ME). Images were captured using a high-speed camera (1000 frames/s) (MiCAM Ultima-L, SciMedia) to generate activation maps and calculate conduction velocities. Atria arrhythmia inducibility was assessed and characterized as described above. Collected images were stored using MiCAM Ultima Experiment Manager (Brainvision) and processed using BV_Analyze (Brainvision, Tokyo, Japan). Activation maps were generated of continuous (4 seconds) optical recordings and used to calculate conduction velocity using the MATLAB-based ElectroMap electrophysiology mapping software **(10)**.

Histology and immunohistological staining: After functional assessments were completed, animals were weighed and euthanized with an anesthetic overdose of isoflurane (~5%). After deep anesthesia was achieved, the thorax was opened via a complete bilateral thoracotomy, the inferior vena cava cut, and hearts were perfused with PBS containing 1% KCl followed by 4% PFA in 0.01M PBS administered transapically and stored in 35 mL of 4% PFA in 0.01M PBS overnight at 4°C. Hearts were excised, blotted dry, and weighed, and the atria and ventricular tissue were separated to weigh individual chambers. The right tibia was harvested and measured for heart weight normalization (**Supplementary Table 1**). Next, hearts were washed three times for 1 h

with PBS, cleaned and sagittally cut with a scalpel to reveal the four-chamber view. Individual heart halves underwent sequential washes with increasing concentrations of ethanol (70%, 80%, 95% and 100% x 2) and xylene (xylene-ethanol, xylene x 2) and were placed in paraffin overnight at 60-70°C for embedding in paraffin blocks.

For histology, the paraffin blocks were deparaffinized as previously described **(11)** and were sliced into 5µm thin sections, at three levels (100 µm apart), sections were stained with Picrosirius red (PSR) for collagen visualization and quantification. The atria were imaged using an Aperio AT2 brightfield whole slide scanner (Leica Biosystems, Wetzlar, Germany) (20x; 0.5 µm/pixel) and analysed with Aperio ImageScope. Collagen expression was quantified using ImageJ software (NIH, Bethesda, MD, USA) as the ratio of positively stained tissue area to total tissue area of each section using the threshold method (12), which exploits the brightness of collagen-stained tissue relative to background tissue and expresses collagen as a percentage relative to total tissue pixel counts.

To visualize macrophage infiltration, mouse cardiac macrophages were identified using a rat anti-mouse primary F4-80 antibody (dilution 1:100; RRID: AB_323 806; Bio-Rad, Mississauga, ON, Canada) with secondary goat anti-rat 647 antibodies (dilution 1:100; RRID: AB_141 778; Invitrogen, Burlington, ON, Canada) that have been previously validated in murine heart tissue **(13, 14)**. Wheat germ agglutin (WGA)(dilution 1:100) was used to stain the membrane, and slides were mounted with anti-fade 4'-6-diamidino-2-phenylindole (DAPI)-containing medium (Invitrogen) to stain cell nuclei.

A Nikon A1R (Nikon) confocal laser scanning microscopy system was used to acquire the whole atrium image of each section by combining both XY stitching and Z stack function. To quantify macrophage infiltration, F4/80+ cell counts per mm^2 of tissue area were determined for

each slice, where only cells co-staining for DAPI and F4-80 were considered true macrophages. For ventricular assessments, 5–10 images were randomly sampled from the LV of each section and quantified as above.

Quantitative real-time PCR: Heparinized mice were anesthetized (2.5% isoflurane) and sacrificed via cervical dislocation. Hearts were quickly excised into cold phosphate-buffered saline (PBS) to inhibit protein and RNA degradation and cell death. Whole hearts were weighed, dissected into left atrial appendage (LAA) and left ventricular (LV) free wall, and each chamber was weighed and flash-frozen in liquid nitrogen to preserve RNA integrity. Frozen tissue (atria and left ventricular free wall) was sonicated in 500µl TRIzol reagent (Invitrogen, 15596026). Lysates were centrifuged, and the resulting supernatant was combined with 100 µl of chloroform for phase separation. The upper aqueous layer was isolated, mixed with 300 µL of isopropanol to precipitate the RNA, and then transferred to silica-based RNA binding columns. RNA purification was completed using the Aurum™ RNA Mini Kit (Bio-Rad, #7326820) according to the manufacturer's instructions.

RNA concentration and purity were assessed using a Nanodrop™ One Microvolume UV-Vis Spectrophotometer (Thermo Fisher Scientific). Reverse transcription was performed using the High-Capacity cDNA Reverse Transcription Kit (Applied Biosystems, #4368814) with 500ng total RNA per reaction.

Relative mRNA transcript levels (normalized to GAPDH) were determined with quantitative real-time PCR using PowerUp™ SYBR™ Green Master Mix (Applied Biosystems, #A25741) on the CFX Opus 96 Real-Time PCR System (BIO-RAD – 12011319). Each 10 µL reaction contained 10 ng cDNA, 5µl SYBR and 500nm of both forward and reverse primers and

run for 40 cycles at 60ºC. GAPDH was used as the housekeeping gene. The expression levels were measured using the ΔΔCT method, and data were reported as fold change.

The following primer sequences were used: *Lox*, 5’- CAAGGGACATCGGACTTCTTA-3’ (forward), 5’-TGGCATCAAGCAGGTCATAG-3’ (reverse); *Loxl1*, 5’-GCCAGTGGATCGACATAACTG-3’ (forward), 5’-ACAATGTACTTGGGGTTCACG-3’ (reverse); *Loxl2*, 5’- TGACTGCCAGTGGATAGACATC-3’ (forward), 5’-GTTGGGGTTAATGACAACCTG-3’ (reverse); *Loxl3*, 5’- CTACTGCTGCTACACTGTCTGT-3’ (forward), 5’- GACCTCATAGGGCTTTCTAGGA-3’ (reverse); *Loxl4*, 5’-TGCCGCTGCAAGTATGATG-3’ (forward), 5’- TGTTCCTGAGACGCTGTTCC-3’ (reverse); *Col1a1*, 5’- TTTGGATGGTGCCAAGGGAG-3’ (forward), 5’-CACCATCATTTCCACGAGCA-3’ (reverse); *Col3a1*, 5’- AAGGCTGCAAGATGGATGCT-3’ (forward), 5’- AAGGCTGCAAGATGGATGCT-3’ (reverse); *Col4a1*, 5’-ATGGCTTGCCTGGAGAGATAGG-3’ (forward), 5’- TGGTTGCCCTTTGAGTCCTGGA-3’ (reverse); *Fn1*, 5’- GGGAGGAAGAAGACAGATGAG-3’ (forward), 5’-TACCCAGGGTTGGTGATGAA-3’ (reverse); *Tgfb*, 5’-GGAATACAGGGCTTTCGATT-3’ (forward), 5’- CTCTGTGGAGCTGAAGCAAT-3’ (reverse); *Mmp2*, 5’-GATACCCTCAAGAAGATGCAGAAGT-3’ (forward), 5’-ATCTTGGCTTCCGCATGGT-3’ (reverse); *Mmp9*, 5’- AATCTCTTCTAGAGACTGGGAAGGAG-3’ (forward), 5’-AGCTGATTGACTAAAGTAGCTGGA-3’ (reverse); *Nppa*, 5’-TGATGGATTTCAAGAACCTGCT-3’ (forward), 5’- TCTCAGAGGTGGGTTGACCT-3’ (reverse); *Nppb*, 5’-TTTGGGCTGTAACGCACTGA-3’ (forward), 5’-TCTCAGAGGTGGGTTGACCT-3’ (reverse); *Tnf*, 5’- CCCACGTCGTAGCAAACCA-3’ (forward), 5’-ACAAGGTACAACCCATCGGC-3’ (reverse); *Il1b*, 5’-

CAACCAACAAGTGATATTCTCCATG-3’ (forward), 5’- GATCCACACTCTCCAGCTGCA-3’ (reverse); *Il6*, 5’-GAGGATACCACTCCCAACAGACC-3’ (forward), 5’-AAGTGCATCATCGTTGTTCATACA-3’ (reverse); *Eln*, 5’-CAGGAGTTAGGCTCCCAGGT-3’ (forward), 5’- TCCACCTCTGGCTCCGTATT-3’ (reverse); *Myh7*, 5’- TACTTGCTACCCTCAGGTGGCT-3’ (forward), 5’-GGTAAGCCCAGGCCTGTAGA-3’ (reverse); *Gapdh*, 5’- TGCACCACCAACTGCTTAG-3’ (forward), 5’- GGATGCAGGGATGATGTTC-3’ (reverse).

Sex as a biological variable: Our study exclusively examined male mice because male mice exhibited less phenotypic and technical variability. Moreover, clinically men have been shown to have a higher prevalence of moderate-to-severe AR compared to women **(15-17)**. Nonetheless, a preliminary pilot study suggests the findings are relevant for female mice.

Statistics: Data are presented as mean±SEM. Continuous variables were compared between group (Sham versus AR) and/or chamber (atria versus ventricle) with 2-tailed Student’s *t*-tests or a two-way ANOVA with subsequent pairwise comparisons adjusted with the Sidak’s multiple-comparison approach. Homogeneity of variance was assessed using Levene’s test. AF durations were assessed using a Mann Whitney U test with Dunn’s multiple comparison test as the data were not normally distributed (D’Agostino & Pearson omnibus normality test). To compare arrhythmic events, a 2x2 contingency table with Fisher’s exact test was used. A repeated measure two-way ANOVA with Sidak’s multiple-comparison test was used to analyze changes in end-systolic and end-diastolic pressures as well as derivatives of maximal and minimal changes in pressure with changes in time within and between Sham and AR telemetry-implanted mice. To determine the

relationship between AR severity (VTI_{ratio}) and outcome variables, a linear regression ordinary least squares (OLS) or generalized linear model (GLM) was used. For the relationship between VTI_{ratio} and arrhythmia durations, after visual inspection of the data with a scatterplot, a GLM with a gamma distribution and log link function was used to assess the association between VTIratio and atrial arrhythmia durations for both atria and ventricles. A gamma distribution was deemed appropriate because the atrial arrhythmia duration is strictly positive (Y > 0 seconds) and variance in Y increased with the mean of Y, with the log link ensuring all predictions remain strictly positive and models for a curvilinear (multiplicative) relationship between X and Y. P values < 0.05 were considered statistically significant. All statistical analyses were carried out using GraphPad Prism (GraphPad Software, Inc).

Study approval: This study was carried out in adherence with the current guidelines of the Canadian Council on Animal Care and National Institutes of Health (NIH). The protocol was approved by the Animal Care Committee at York University (2019-14).

Data availability: All data in this study are available in the Supporting Data Values file as well as from the corresponding author upon reasonable request.

Acknowledgements

None

Funding Support: This work was supported by Canadian Institutes of Health Research (CIHR) grant MOP-125950 and Canada Research Chair in Cardiovascular Biology (to PHB); a Canadian Foundation for Innovation John Evans Leader Award (to PHB); and a CIHR PDF (to RL).

Author Contributions

R.L. and P.H.B. conceptualized the study. R.L., X.L., D.S, R.D., N.P., and J.W. collected the data. R.L. and M.D. analyzed the data, and R.L. prepared the manuscript. P.H.B supervised the work.

REFERENCES

1. Wu J, et al. Left ventricular response in the transition from hypertrophy to failure recapitulates distinct roles of Akt, β-arrestin-2, and CaMKII in mice with aortic regurgitation. *Annals of translational medicine.* 2020;8(5):219.
2. Otto CM, et al. 2020 ACC/AHA Guideline for the Management of Patients With Valvular Heart Disease: A Report of the American College of Cardiology/American Heart Association Joint Committee on Clinical Practice Guidelines. *Circulation.* 2021;143(5):e72-e227.
3. Lakin R, et al. Differential negative effects of acute exhaustive swim exercise on the right ventricle is associated with disproportionate hemodynamic loading. *Am J Physiol Heart Circ Physiol.* 2021.
4. Aschar-Sobbi R, et al. Increased atrial arrhythmia susceptibility induced by intense endurance exercise in mice requires TNFalpha. *Nat Commun.* 2015;6:6018.
5. Heinen A, et al. Echocardiographic Analysis of Cardiac Function after Infarction in Mice: Validation of Single-Plane Long-Axis View Measurements and the Bi-Plane Simpson Method. *Ultrasound Med Biol.* 2018;44(7):1544-55.
6. Lakin R, et al. Inhibition of soluble TNFalpha prevents adverse atrial remodeling and atrial arrhythmia susceptibility induced in mice by endurance exercise. *J Mol Cell Cardiol.* 2019;129:165-73.
7. Tuomi JM. *Physiology*. Scholarship@Western: The University of Western Ontario; 2011:209.
8. Lakin R, et al. Cardiomyocyte and endothelial cells play distinct roles in the tumour necrosis factor (TNF)-dependent atrial responses and increased atrial fibrillation vulnerability induced by endurance exercise training in mice. *Cardiovasc Res.* 2023;119(16):2607-22.
9. Li T, Sperelakis N, Teneick RE, and Solaro RJ. Effects of diacetyl monoxime on cardiac excitation-contraction coupling. *J Pharmacol Exp Ther.* 1985;232(3):688-95.
10. O'Shea C, et al. ElectroMap: High-throughput open-source software for analysis and mapping of cardiac electrophysiology. *Sci Rep.* 2019;9(1):1389.

11. Gorman RA, et al. The effects of daily dose of intense exercise on cardiac responses and atrial fibrillation. *J Physiol.* 2024;602(4):569-96.
12. Hadi AM, et al. Rapid quantification of myocardial fibrosis: a new macro-based automated analysis. *Cell Oncol (Dordr).* 2011;34(4):343-54.
13. Mai H, et al. Whole-body cellular mapping in mouse using standard IgG antibodies. *Nat Biotechnol.* 2024;42(4):617-27.
14. Tamaki S, et al. Interleukin-16 promotes cardiac fibrosis and myocardial stiffening in heart failure with preserved ejection fraction. *PLoS One.* 2013;8(7):e68893.
15. Singh JP, et al. Prevalence and clinical determinants of mitral, tricuspid, and aortic regurgitation (the Framingham Heart Study). *Am J Cardiol.* 1999;83(6):897-902.
16. DesJardin JT, et al. Sex Differences and Similarities in Valvular Heart Disease. *Circ Res.* 2022;130(4):455-73.
17. Mascherbauer J, et al. Sex-related differences in severe native valvular heart disease: the ESC-EORP Valvular Heart Disease II survey. *Eur Heart J.* 2024;45(37):3818-33.

SUPPLEMENTAL FIGURE

Figure Caption

Figure S1. Graded aortic regurgitation (AR) induces ventricular remodeling without increasing ventricular arrhythmia vulnerability. (A) Kaplan-Meier curve showing a 90% (20/22) survival in all mice subjected to aortic regurgitation (AR) and 100% (19/19) in Sham-operated mice. AR mice were euthanized due to post-operative clot formation (n=1) or VTIratio ≥60% (n=1). **(B)** Representative initial 24-hour (acute) post-op implanted telemetry-derived hemodynamic tracking of the derivatives of the maximal and minimal rates of left ventricular (LV) pressure changes over time during isovolumic contraction (dP/dt_{max}) and isovolumic relaxation (dP/dt_{min})(bottom panels) in Sham (red) and AR (black) mice; light:dark cycles indicated. **(C)** Representative 4-week Sham and AR implanted telemetry-derived hemodynamics. Light:dark cycles indicated, AR mice show reduced lusitropic reserve and increased filling pressures during activity (dark cycle). **(D)** Changes in echocardiographically- and hemodynamically-derived LV structural and functional indices. AR mice show gradual LV dilatation (increased end-systolic and end-diastolic diameters) and functional decline (ejection fraction and $dPdt_{min}$) over 4-weeks in response to AR. **(E)** Ventricular arrhythmia durations were increased (P=0.055) in AR versus Sham mice, with arrhythmia durations increasing as a function of the degree of regurgitation (VTI_{ratio}) and the nature of the relationship to LV decompensation. **(F)** Ventricular weights-to-tibial length were increased in AR (n=12) compared to SHAM (n=10) mice, with the degree of hypertrophy increasing as a function of VTI_{ratio}. **(G-H)** Bright field images of picrosirius red (PSR) stained LV sections. Quantification of collagen deposition (%) show no significant elevation in LV fibrosis in AR (n=6) compared SHAM (n=5) mice. However, the degree of LV fibrosis increased as a function of the degree of aortic regurgitation (VTI_{ratio}). **(I-J)** Confocal micrographs of LV sections stained with F4/80+ to quantify macrophage infiltration (arrows) in 4-week SHAM and AR mice. No significant difference in F4/80+ cell counts were observed between groups. Data presented as mean ± SEM. *P* values from Student's *t-test*. ***P < 0.001; NS, not significant.

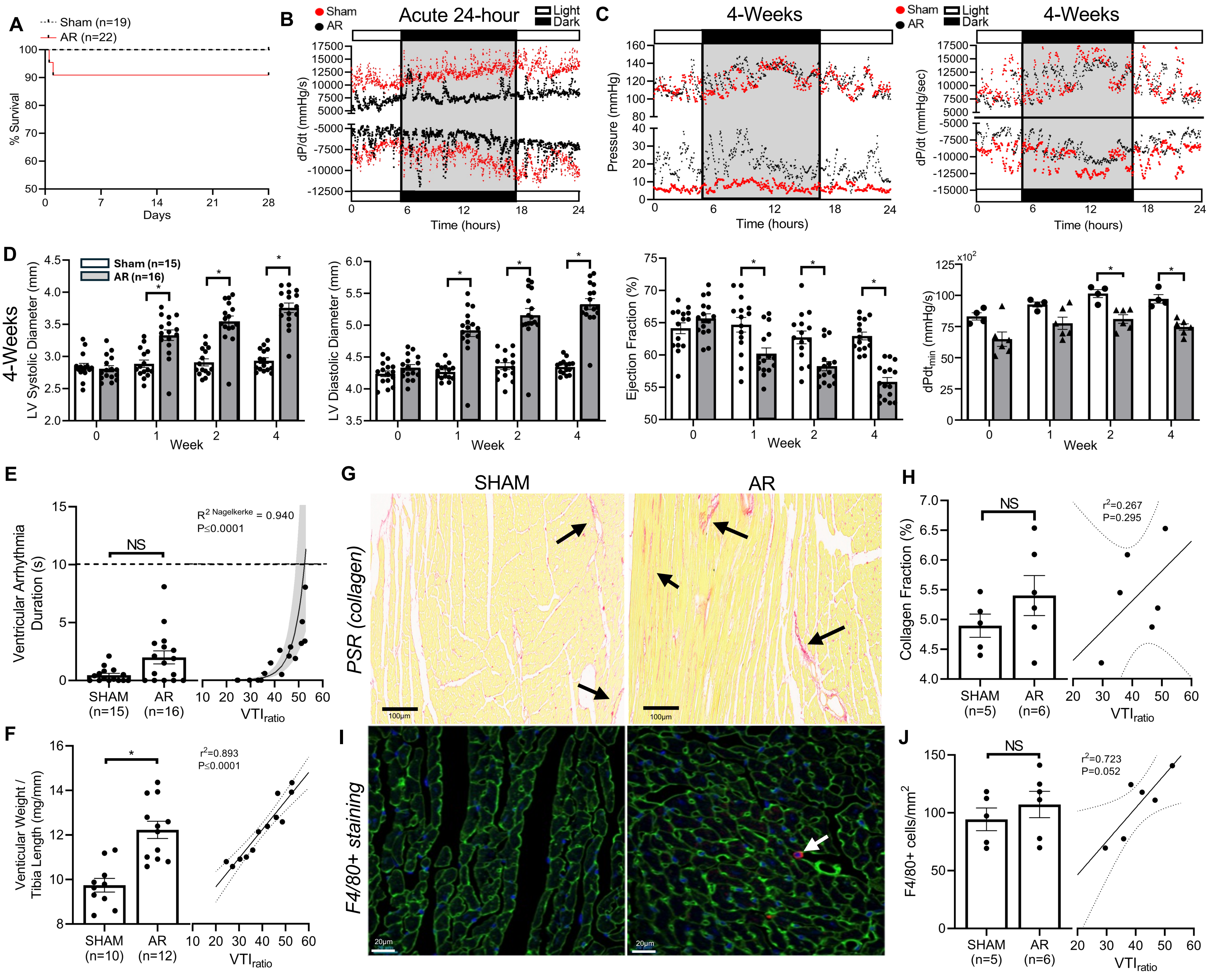

A
Sham (n=19)
AR (n=22)
% Survival
Days
B
Sham
AR
Acute 24-hour
Light
Dark
dP/dt (mmHg/s)
Time (hours)
C
4-Weeks
Pressure (mmHg)
dP/dt (mmHg/sec)
D
4-Weeks
Sham (n=15)
AR (n=16)
LV Systolic Diameter (mm)
LV Diastolic Diameter (mm)
Ejection Fraction (%)
dPdt min (mmHg/s)
x10^2
Week
E
Ventricular Arrhythmia Duration (s)
NS
R^2 Nagelkerke = 0.940
P≤0.0001
SHAM (n=15)
AR (n=16)
VTI ratio
F
Venticular Weight / Tibia Length (mg/mm)
r^2=0.893
P≤0.0001
SHAM (n=10)
AR (n=12)
G
SHAM
AR
PSR (collagen)
100μm
H
Collagen Fraction (%)
r^2=0.267
P=0.295
SHAM (n=5)
AR (n=6)
I
F4/80+ staining
20μm
J
F4/80+ cells/mm^2
r^2=0.723
P=0.052

Supplemental Table 1

	Sham	Aortic Regurgitation	P-value
**Morphometry *n***	**15**	**16**	
**Body weight (g)**	45.4±1.1	43.7±1.1	0.3100
n	**10**	**12**	
**HW/TL (mg/mm)**	10.6±0.3	13.5±0.4*	≤0.0001
**AW/VW (mg/g)**	92.6±2.0	101.8±4.9	0.1149
**Echocardiography *n***	**15**	**16**	
**Heart rate (bpm)**	530±15	517±11	0.9401
**Stroke Volume (µl)**	55.8±1.2	76.1±3.0*	≤0.0001
**Fractional Shortening (%)**	34.5±0.4	29.9±0.7*	≤0.0001
**Posterior Wall Thickness (mm)**	0.794±0.004	0.900±0.009*	≤0.0001
**Hemodynamics *n***	**4**	**6**	
**LV End-Systolic Pressure (mmHg)**	113.4±1.9	124.6±4.4	0.1950
**$dPdt_{max}$ (mmHg/s)**	11,875±917	9,734±316	0.3994
**$dPdt_{min}$ (mmHg/s)**	9,727±343	7,470±254*	0.0088
**Electrophysiology *n***	**15**	**16**	
**Ventricular ERP (ms)**	28.5±0.8	29.2±0.8	0.5338
**Atrioventricular ERP (ms)**	50.6±1.3	52.1±1.5	0.4472

AW, atrial weight; $dPdt_{max}$, maximum rate of left ventricular pressure increase during isovolumic contraction; $dPdt_{min}$, maximum rate of left ventricular pressure decrease during isovolumic relaxation; HW, heart weight; TL, tibia length; VW, ventricular weight

Supplemental Table 2: Left atrial qPCR mRNA expression

Gene	1W SHAM	1W AR	P-Value	4W Sham	4W AR	P-Value
LOXL1	1.089±0.171 (7)	1.400±0.161 (4)	0.2594	1.100±0.281 (4)	1.461±0.195 (4)	0.3323
LOXL2	0.787±0.0638 (10)	1.311±0.223 (10)*	0.0336	1.154±0.239 (8)	0.8262±0.120 (10)	0.3599
LOXL3	1.009±0.218 (10)	1.568±0.435 (10)	0.3527	1.059±0.216 (8)	1.313±0.170 (10)	0.3154
LOXL4	0.9381±0.141 (10)	0.9375±0.144 (10)	0.7959	1.089±0.182 (8)	0.992±0.0976 (10)	0.6965
LOX	1.158±0.315 (6)	1.551±0.2838 (6)	0.3095	1.115±0.206 (5)	1.306±0.275 (6)	0.6623
COL I	0.9694±0.197 (10)	2.196±0.484 (10)*	0.0232	1.264±0.364 (5)	0.9292±0.249 (6)	0.4286
COL III	1.010±0.117 (10)	3.018±0.891 (10)	0.1655	1.380±0.382 (8)	1.238±0.209 (10)	0.9654
COL IV	1.107±0.174 (10)	0.9846±0.173 (10)	0.8534	0.561±0.161 (3)	0.628±0.162 (4)	0.8571
MMP2	1.137±0.158 (10)	1.137±0.125 (10)	0.7959	1.188±0.191 (8)	0.821±0.0836 (10)	0.0831
MMP9	2.743±1.188 (7)	1.416±1.320 (4)	0.2303	1.316±0.265 (8)	1.082±0.361 (10)	0.3154
MMP9/2	2.459±0.995 (6)	13.57±5.852 (4)	0.1714	1.175±0.245 (9)	3.760±1.615 (10)	0.9682
FN1	1.093±0.221 (10)	1.328±0.253 (10)	0.4813	1.185±0.220 (7)	1.799±0.529 (10)	0.6009
ELN	1.144±0.281 (6)	2.373±0.781 (6)	0.3095	1.328±0.425 (5)	1.930±0.606 (6)	0.6623
TGF-β	1.256±0.393 (10)	1.814±0.595 (10)	0.6305	1.199±0.173 (8)	0.460±0.120 (10)*	0.0062
Nppa	1.025±0.106 (6)	1.589±0.554 (6)	0.4848	3.111±0.913 (5)	2.498±0.558 (6)	0.6623
Nppb	1.041±0.121 (6)	1.408±0.265 (6)	0.5887	1.809±0.687 (5)	1.953±0.320 (6)	0.6623
MHY7	1.600±0.498 (6)	1.779±0.966 (6)	0.8182	1.295±0.454 (5)	2.496±1.002 (5)	0.6905
TNFa	1.182±0.275 (6)	0.885±0.170 (6)	0.6991	2.639±1.574 (5)	0.355±0.086	0.2468
IL-6	1.575±0.659 (6)	0.997±0.262 (6)	0.9372	2.422±1.233 (5)	0.8542±0.185 (6)	0.6623
IL-1β	1.178±0.283 (6)	0.5662±0.158 (6)	0.0931	1.978±0.904 (5)	1.003±0.378 (6)	0.6623

Col I/III/IV, collagen I/III/IV; ELN, elastin; FN1, fibronectin 1; IL-6, interleukin-6; IL-1β, interleukin-1 beta; LOX, lysyl oxidase; LOXL1/2/3/4, LOX-like enzyme 1/2/3/4; MHY7, beta-myosin heavy chain; MMP2/9/14, matrix metallopeptidase 2/9; Nppa, atrial natriuretic peptide; Nppb, B-type natriuretic peptide; TGF-β, transforming growth factor β; TNFa, tumor necrosis factor-alpha

Supplemental Table 3: Left ventricular qPCR mRNA expression

Gene	1W SHAM	1W AR	P-Value	4W Sham	4W AR	P-Value
LOXL1	1.035±0.104 (7)	2.281±0.583 (4)*	0.0242	1.051±0.164 (5)	0.494±0.127 (5)	0.4776
LOXL2	1.068±0.0943 (10)	1.643±0.311 (10)	0.1230	1.024±0.160 (5)	0.742±0.0684 (6)	0.0823
LOXL3	1.068±0.116 (10)	1.381±0.206 (10)	0.1903	0.993±0.118 (5)	0.913±0.117 (6)	0.6623
LOXL4	1.052±0.119 (10)	1.298±0.171 (10)	0.1903	0.997±0.121 (5)	0.954±0.158 (6)	0.9999
LOX	1.056±0.155 (6)	1.840±0.685 (6)	0.4848	1.033±0.129 (6)	1.232±0.353 (6)	0.8182
COL I	1.050±0.155 (6)	4.247±2.223 (4)	0.4762	1.047±0.163 (5)	0.775±0.247 (5)	0.7125
COL III	1.108±0.230 (7)	2.270±0.419 (4)*	0.0424	1.033±0.136 (5)	0.566±0.077 (5)	0.4095
COL IV	1.019±0.0913 (6)	0.9886±0.127 (6)	0.9372	1.042±0.167 (5)	0.789±0.098 (5)	0.9323
MMP2	1.050±0.141 (7)	1.532±0.0516 (4)	0.1091	1.039±0.140 (5)	0.513±0.074 (5)	0.3186
MMP9	1.104±0.112 (10)	0.5820±0.104 (10)*	0.0052	1.141±0.244 (5)	0.879±0.144 (6)	0.6623
FN1	1.015±0.0773 (10)	2.230±0.398 (10)*	0.0288	1.088±0.223 (5)	1.193±0.188 (6)	0.6623
ELN	1.008±0.0559 (6)	1.318±0.169 (6)	0.1797	1.219±0.345 (6)	1.097±0.101 (6)	0.4848
TGF-β	1.046±0.122 (9)	0.8596±0.153 (10)	0.4002	0.978±0.109 (5)	1.131±0.105 (6)	0.9307
Nppa	1.201±0.344 (6)	4.410±0.865 (6)*	0.0152	1.087±0.182 (5)	4.243±1.395 (6)*	0.0087
Nppb	1.032±0.107 (6)	2.011 ±0.103 (6)*	0.0022	1.050±0.151 (6)	4.477±0.682 (6)*	0.0022
MHY7	2.129±1.042	0.677±0.318	0.3095	1.047±0.135	3.939±1.630	0.0649
TNFa	1.007±0.0510 (6)	1.093±0.0728 (6)	0.2403	1.484±1.091 (5)	0.557±0.182 (6)	0.7922
IL-6	1.031±0.130 (5)	1.727 ±0.298 (6)	0.0519	1.917±0.870 (6)	0.651±0.0656 (6)	0.4848
IL-1β	1.056±0.193 (5)	0.5433±0.096 (6)*	0.0303	1. 810±0.864	0.999±0.487	0.6991

Col I/III/IV, collagen I/III/IV; ELN, elastin; FN1, fibronectin 1; IL-6, interleukin-6; IL-1β, interleukin-1 beta; LOX, lysyl oxidase; LOXL1/2/3/4, LOX-like enzyme 1/2/3/4; MHY7, beta-myosin heavy chain; MMP2/9/14, matrix metallopeptidase 2/9; Nppa, atrial natriuretic peptide; Nppb, B-type natriuretic peptide; TGF-β, transforming growth factor β; TNFa, tumor necrosis factor-alpha
